# Is cloning horses ethical?

**DOI:** 10.1111/eve.12566

**Published:** 2016-03-07

**Authors:** M. L. H. Campbell

**Affiliations:** ^1^ Department of Production and Population Health The Royal Veterinary College North Mymms Herts UK

**Keywords:** horse, equine cloning, welfare, ethics, equine assisted reproduction

## Abstract

This paper assesses whether cloning horses is ethical by reviewing ethical arguments against cloning of nonequine species and determining whether they apply to horses, analysing ethical arguments about horse cloning which do not apply to noncompetitive species and considering the ethical dilemmas faced by veterinarians involved in horse cloning. The author concludes that concerns about the health and welfare of cloned horses render the technique ethically problematic and that the onus is on those providing commercial equine cloning services to collate data and provide a stronger evidence base for ethical decision‐making.

## Introduction

Somatic cell nuclear transfer (SCNT) or ‘cloning’ is currently being offered as a commercial method of horse reproduction in countries including the European Union, the United States, Australia, New Zealand and South America (Hinrichs 2005, 2006; Herrera 2015; Hinrichs and Choi 2015). In 2012, it was estimated that there were 100–200 cloned horses worldwide (Hinrichs 2012). It is likely that the number of cloned horses being born per year is small. Herrera (2015) reported that 20 viable cloned foals had been produced over 4 years in South America, whilst Reis (2015) estimated that 2–5 cloned foals per year are born in Europe.

Cloning is not allowed by international studbooks registering racing Thoroughbreds. The American Quarter Horse Association is another notable example of a studbook which refuses to register clones (http://www.latimes.com/nation/la-na-cloned-horses-20150314-story.html). Studbooks which will register clones include the majority of Warmblood stud books and the World Breeding Federation for Sport Horses. It is probable that cloning has been used in horses of various breeds being used for the disciplines regulated by the Federation Equestrian Internationale (FEI), although the FEI does not have data on which horses competing under FEI Regulations are clones (G. Akerstrom, personal communication).

Reasons why owners choose to clone horses include the production from a competitively successful castrated male animal of an entire male clone which can be used for breeding, the attempted ‘recreation’ of a favourite animal and attempted duplication of a successful competition horse.

Cloning of any species of animal is ethically contentious (Nolen 2007). In 2012 the FEI changed its rules to allow clones and offspring of clones to compete. However, the European Commission in December 2013 tabled proposals to ban the use of the cloning technique in the EU for farm animals and the import of such animal clones (IP/13/1269 18/12/2013). The proposals of the European Commission included horses used for agricultural production purposes, but allowed derogations for ‘…animals kept and reproduced exclusively for other purposes such as research, the production of medicinal products and medical devices, the preservation of rare breeds or endangered species, sporting and cultural events’, which effectively excluded horses used for purposes other than agricultural production from the proposed ban on cloning. In October 2015, the European Parliament amended the European Commission's proposal, to remove the derogation from the ban for sporting and cultural events (Amendment 30: http://www.europarl.europa.eu/sides/getDoc.do?type&TA%26language&EN%26reference&P8-TA-2015-0285). This means that should the European Parliament's amendments be agreed in regulation negotiations with the European Council, cloning of all horses except those of endangered breeds for which no other method of reproduction can be used will be banned in the EU. However, cloning of horses for all purposes will continue to be allowed in many other countries.

Against this rather incoherent legislative background, the aim of this article is to determine whether there are convincing ethical arguments for banning cloning of horses. It first reviews general ethical arguments against the cloning of animals of all species and analyses whether they apply to horses. Are arguments against cloning of other species convincing? If so, should they apply to horses, or is there something different about cloning horses which could make cloning horses ethical even if cloning other animals is not so? Secondly, the article considers whether there are any ethical arguments against cloning which apply to horses but not to other species, for example, arguments about sporting ethics. Finally, ethical dilemmas which might face veterinarians who are asked by their clients to become involved in equine cloning are presented.

## Food safety concerns

In parts of Europe, although not commonly in the UK, horses are eaten. The initial debate within the European Union about cloning of farm animals incorporated concern about the possible health effects on man of eating cloned animals and their products. Were these concerns justified and do they apply to horses?

There is undoubtedly public concern about the health implications of consuming clones or their products (Anon 2008a,b, Aizaki *et al*. 2011). Scientific evidence suggests that such concerns are unfounded (Anon 2008b, 2009a, 2010, 2012a). Although this evidence relates to ruminants rather than to horses, it is hard to see why health risks in man should be associated with eating cloned horsemeat when there are none associated with eating cloned meat derived from other species. The European Commission has indicated that a ban on cloning food‐producing animals is not justified on food safety grounds (Anon 2012a) and there is no evidence that this does not apply to horses as well as ruminants.

In the USA, Argentina and Brazil, unlabelled cloned animals and their products are now allowed in the food chain and can be exported (Anon 2012b). Cloned meat is primarily beef, but no data is available on the consumption of cloned horsemeat. Some consumers feel that it is unethical to clone animals even if eating cloned produce is safe (see below). Cloned meat entering the food chain unlabelled does therefore raise an ethical issue about transparency, relating to consumers’ rights to know what they are eating. This would apply equally to horsemeat or other types of meat being produced by cloning.

## Is cloning animals simply morally wrong?

There is undeniably something fundamentally different about cloning compared with all other assisted reproductive techniques (ARTs), since cloning aims to reproduce an existing animal, whereas all other ARTs aim to produce a novel animal. The idea that there is something fundamentally wrong with cloning, that it is somehow beyond the realms of moral acceptability, pervades the literature on cloning in man (Shapiro 1996; Petersen 2002) and is often expressed as ‘an affront to human dignity’. Such concerns are voiced even in institutional documents (Harris 1997) such as the Oviedo Convention for the Protection of Human Rights and Dignity of the Human Being with regard to the Application of Biology and Medicine (Harris 1997) and the Report of the President's Council on Bioethics: Human Cloning and Human Dignity (2002). These concerns quite probably do reflect public sentiment, but are poorly defined (Harris 1997; Savulescu 2005; Simpson 2007).

Interestingly, there is a similarly poorly defined public repugnance with the concept of cloning animals, which might be characterised as an ‘affront to the animal's dignity’. Thus one investigation into public attitudes to animal cloning (Gjerris *et al*. 2006) found that ‘moral assessment is the most important factor behind the level of support’; that people were concerned about ‘violation of the integrity of animals that cloning might constitute’ and that ‘cloning, seems to cross an invisible border between the natural and the unnatural’. In the 2008 Eurobarometer survey (Anon 2008a,b), 61% of respondents thought that animal cloning was ‘morally wrong’ and 38% said that animal cloning was unethical on moral grounds, whatever the potential benefits in medical or food production terms.

Such moral objections to cloning would apply equally to all food‐producing animals, including horses. Yet these moral objections are no better defined for animals than they are for man. Most ARTs violate nature, yet the public seem only really to object to cloning: embryo transfer; artificial insemination and IVF do not inspire a similarly visceral response. Gjerris *et al*. (2006) suggests that this is because ‘although the concept of naturalness leaves many questions to be answered, it is (undoubtedly) contradicted by the asexual character of reproduction by cloning’. Certainly, although asexual reproduction does occur in nature, asexual reproduction of farm animals at least (for which the most data on public opinion exists) does not.

Whilst public unease about animal cloning based on ill‐defined notions of ‘dignity’ undeniably exists, such unease is not necessarily a strong ethical reason to ban cloning, either of horses or of any other species. ‘..moral gut responses may be morally admirable but they may also be morally wrong’ (Anon 2009b). Whilst the concept of (inviolable) human dignity pervades religion, medicine and law, there is no proof that animals themselves have any concept of ‘dignity’.^1^ We might consider that there is nothing dignified about an animal kept under an intensive farming system being used for medical research, or being carried in Paris Hilton's handbag, but an ‘affront to dignity’ seems a weak ethical reason for objecting to any of these practices, all of which can legitimately be objected to for other stronger reasons relating to failure to meet an animal's welfare needs.

## Cloning for conservation

The draft legislation to ban cloning of farm animals proposed by the European Commission in 2013 and amended by the European Parliament in 2015 includes exceptions for the conservation of rare breeds and endangered species. Where animal numbers are small and animals are wild or semi‐feral, making the application of invasive ARTs difficult, cloning may provide a method of preserving rare genetic material and promoting biodiversity (Anon 2009c; Yang *et al*. 2010). Might cloning of horses to preserve rare breeds, for example in the face of an outbreak of fatal exotic disease, such as African horse sickness, be ethically justifiable even if cloning for other, competitive or sentimental purposes was considered unethical?

The welfare costs of cloning are discussed in the next section. These costs to individual animals are independent of the reason(s) for undertaking cloning. Any ethical justification for allowing cloning to facilitate conservation would therefore have to be based in a cost:benefit argument that any costs to individual animals are outweighed by a perceived benefit of preserving the species. Yet is there an absolute benefit in species preservation? Is biodiversity necessarily a good thing? Is the loss of some species (or in the case of horses, breeds) not simply a normal Darwinian mechanism?

The consequences of cloning to increase or preserve biodiversity could themselves have ethical implications; cloning a woolly mammoth, for example, is now feasible, but the consequences in terms of impact on the environment, habitat, other animals and on the welfare of the animal itself (reintroduced to an environment so different from the one which its species last inhabited) are unknown. Whilst it may seem a subjective shame to lose some of the equine rare breeds, the argument that they perform an environmental function which no other breed can do is tenuous. Indeed the environmental function of some rare breeds, such as the Suffolk Punch, has decreased significantly with the mechanisation of agriculture. Even the management of moorlands, to which the rare breed of Exmoor ponies, for example, make a significant contribution, can nowadays be undertaken by other methods. Thus, although governments may face legal obligations under the United Nations Convention on Biological Diversity (1992) to promote biodiversity, the ethical imperative to do so by subjugating the welfare of individual animals to the desire to preserve an equine breed is unconvincing.

## Are there welfare‐based reasons which make animal cloning unethical?

Current cloning techniques result in recognised welfare problems (Renard *et al*. 2001; Houdebine *et al*. 2008; Anon 2012b; Kim *et al*. 2012, 2014). Problems associated with cloning across a variety species, but particularly cattle, include placental abnormalities during pregnancy, fetal abnormalities, dystocia related to ‘large offspring syndrome’ in farm animals, neonatal weakness/disease, systemic illness/disease in cloned animals and premature ageing of adult clones (Kuhholzer‐Cabot and Brem 2002; Chavatte‐Palmer *et al*. 2003; Loi *et al*. 2006; Arnold *et al*. 2008; Houdebine *et al*. 2008; Jang *et al*. 2010; Anon 2012b).

One of the prime factors behind the European Commission's decision in late 2013 to propose a ban on the cloning of food animals (http://europa.eu/rapid/press-release_IP-13-1269_en.htm. Accessed 20.03.14) and the recent European Parliament decision to extend that ban (Anon 2015) is the fact that farm animal cloning is viewed as a risk to animal welfare (Gamborg *et al*. 2005). This was made clear by Renate Sommer, German MEP, who stated that the prohibition was based in concerns about ‘the negative effects on animal welfare’ and that ‘prohibiting cloning is a matter of European values and principles’ (Anon 2015). If concerns about the health and welfare of clones and their dams provide a convincing ethical argument against cloning of farm animals, does the same argument apply to horses?

Data about the health and welfare of equine clones is comparatively lacking. Only the groups led by Katrin Hinrichs in the USA (Hinrichs 2006; Johnson *et al*. 2010) and Cesare Galli in Italy (Galli *et al*. 2003; Lagutina *et al*. 2005) have published data on their success rates and on problems associated with current cloning techniques. These data are reviewed in the next few paragraphs.

### Embryo loss in equine cloning

Hinrich's group (Johnson *et al*. 2010) reported that 26% of cloned embryos transferred by them resulted in the birth of a live foal, whereas Galli and coworkers reported that three live foals resulted from transfers of more than 100 cloned embryos (Galli *et al*. 2003; Lagutina *et al*. 2005). In a retrospective study of all nuclear transfer derived embryos at Texas A&M University from 2004 to 2007, Johnson *et al*. (2010) found that 81% of oocytes which had been subjected to nuclear transfer cleaved after activation. Of those cleaved embryos, 5% developed to blastocyst stage. A total of 51% of those blastocysts, having been transferred, resulted in the establishment of pregnancy in the recipient mare, as determined by ultrasonography at 11–16 days post ovulation.

### Fetal abnormalities

The only data available on fetal losses and abnormalities comes from Hinrich's group (Johnson *et al*. 2010). A total of six pregnancies were lost between 3 and 9 months of gestation and one recipient mare developed a neurological condition and was subjected to euthanasia. The cause of the neurological condition was not discovered and her fetus was normal. A total of three fetuses were aborted between 5 and 9 months; one had no abnormalities, one had no detectable abnormalities (it had been scavenged) and one had an umbilical hernia (omphalocoele). One foal was born prematurely and died.

### Dystocia

Data about dystocias in horses is also sparse. Johnson *et al*. (2010) reported that one mare had a dystocia which required caesarean section at full‐term and a dead foal with severe contraction of the forelimbs and an omphalocoele was delivered. A total of 14 of 31 mares which were pregnant between 11 and 16 days post ovulation delivered live foals and 13 were born at normal gestational length. None was oversized or overweight in comparison to the mare and all mares had uncomplicated foalings. One had a prolonged gestation, but was nonetheless born small and underweight; however, at a year of age, its weight was comparable to that of the other surviving foals.

### Neonatal health

In the study of Johnson *et al*. (2010), two foals had signs of hypoxic ischaemic encephalopathy at birth, but recovered with treatment. Two foals died shortly after birth, one from pneumonia and one following anaesthetic complications during an attempt to surgically correct a perceived bladder abnormality. A total of 7 of 14 foals required administration of supplementary oxygen for >12 h after birth, one for 15 days. All foals were treated as ‘high risk’ and given antibiotics after birth. A total of 8 of 14 foals required antibiotics for more than 5 days. Half of the foals had some form of umbilical abnormality, 8/14 had some form of flexural or angular limb deformity (all corrected by 6 months) and 2/14 exhibited incomplete ossification of the cuboidal bones. On average, foals required 8.5 days of intensive care following birth. Those which survived a week grew and developed normally thereafter, apart from one foal which required two surgeries to remove uroliths (bladder stones). Of the three foals produced by Galli and coworkers (Galli *et al*. 2003; Lagutina *et al*. 2005), one died from septicaemia and the other two were reported as being healthy.

It is clear that the evidence base about health and welfare issues associated with cloning using SCNT is much less robust for horses than for farm animal species. The broad summary from the little data available seems to be that equine cloning is associated with high rates of embryonic loss; some incidence of fetal abnormalities (although this appears to be lower than that recorded in farm animals) and a requirement for neonatal intensive care. In contrast to farm animals, dystocia attributable to oversized fetal clones does not seem to be a significant problem in horses. Similarly, the problems of hydrops of the fetal membranes which occur in cloned cattle seem not to occur in mares, possibly due to differences in placentation. It is difficult to know whether some abnormalities in neonatal and foal clones, for example limb deformities, are truly attributable to the cloning process, because such problems are not uncommon in foals anyway. To date, there have been no long‐term studies on the health and welfare of cloned horses.

Further research is clearly needed to increase the evidence base on the short, medium and long‐term health and welfare effects of equine cloning. Until such evidence is available, one could argue that, following the precautionary principle, cloning horses is unethical on welfare grounds, since it seems that there are more problems associated with embryos, fetuses and foals created using SCNT than there are with foals conceived using other equine ARTs.

The caveat to this ethical argument is that many of the problems associated with cloning (in all species) are probably related to technique, particularly *in vitro* culture conditions (van Wagtendonk‐de Leeuw *et al*. 2000). Interestingly, culture conditions which cause large offspring syndrome and abnormal clone phenotype in ruminants, seem not to have the same effects when used for nonruminant embryos (Hill 2014). No data is currently available about any possible correlation between SCNT techniques and particular problems in horses, but it seems reasonable to expect that in horses, as in other species, problems are likely to diminish as techniques improve (Hinrichs and Choi 2015). Paradoxically, techniques will not improve unless cloning continues. This is an argument if equine cloning is to continue to be allowed for those clinics undertaking equine cloning to undertake anonymised and collated reporting on the health and welfare of equine clones at all stages of their lives. Given the small numbers involved, such reporting could operate on a voluntary basis in the equine sector, with due attention to client confidentiality and commercial sensitivities. In order to acquire data about medium and long‐term effects, owner cooperation would be required. Collated reporting would increase the evidence base about health and welfare issues experienced by equine clones and, importantly, any correlation between technique and such problems. Such evidence would simultaneously make it much easier to judge whether on welfare grounds equine cloning is or is not currently ethical and provide an evidence base for improving technique so as to minimise negative effects in future.

## Cloning and sporting ethics

Consideration should be given as to whether there are any additional ethical arguments which might apply to cloning horses but not to cloning other species. One argument around the ethics of cloning which applies to horses and racing dogs, but not to farm animals species, concerns sporting ethics. Although cloned horses have been allowed to compete freely in some disciplines, such as polo, the FEI initially prohibited cloned horses from competing, on the grounds that (i) identifying clones by DNA testing would be problematic, and (ii) cloning conferred a competitive advantage, which violated the spirit of fair play.

There is no convincing argument that cloning is unethical based around identification. Despite concerns that some sports horses are not DNA tested and that the FEI’s ban on cloning was therefore unenforceable (http://internationalanimallaw.com/node/784, accessed 08.01.2016) the vast majority of equine studbooks now use DNA analysis to identify and register horses and would thus be capable of identifying clones and registering them as such. Given that clones do not normally look physically identical to the donor animal or to each other, and that microchipping of horses is commonplace (in some countries, a legal requirement), distinguishing between a cloned and a donor animal or between two clones with identical DNA should not be problematic.

Concerns that cloning confers an unfair competitive advantage are, at the least, unproven. In the one report on racing cloned animals against their noncloned peers, the cloned animals’ performance was mediocre (http://www.thehorse.com/articles/16552/cloned-mules-race-into-history). The FEI does not record clones competing under its rules and does not have data on the competitive success of clones compared with nonclones (G. Akerstrom, personal communication). However, to date, there has been no media coverage reporting that cloned horses have won important FEI events.

There is no convincing evidence that equine cloning is unethical because of reasons relating to sporting ethics. This is consistent with the fact that the FEI reversed its ban on clones competing in 2012.

## Ethical issues facing veterinarians undertaking cloning

Finally, this article considers the ethical dilemmas which might face veterinarians whose clients ask them to become involved in equine cloning. Cloning is a very specialised technique, performed in a small number of centres worldwide. The involvement of most veterinarians is therefore likely to be limited to taking a skin biopsy from a donor animal, to provide the nuclear material necessary for the SCNT process and possibly to providing neonatal and later healthcare for cloned offspring.

All ARTs are ethically unusual in that, unlike most veterinary procedures, they are usually undertaken with no expectation of improving the health or welfare of the animal on which they are performed. In this respect, cloning is no different from commonly used equine ARTs such as artificial insemination and embryo transfer. Depending on the AR technique, the health and welfare of up to three animals (a donor animal, a recipient animal who gestates and gives birth to the foal and the foal itself) should be considered.

In terms of the direct effects on the donor animal, there is nothing inherently ethically different about a veterinarian subjecting a donor animal to a skin biopsy for SCNT than there is about a veterinarian subjecting a donor animal to another ART such as embryo retrieval; both are mildly stressful/painful procedures for which analgesia and sedation can be provided (Campbell and Sandoe 2015), which are not expected to offer any direct benefit to the donor animal. As discussed above, however, cloning is associated with risks to the health and welfare of cloned foals. Such risks have not been proven in foals created by other equine ARTs (Campbell and Sandoe 2015). The risks to foals produced by cloning may provide veterinarians involved in undertaking skin biopsies for SCNT with grounds for questioning the ethical justification of being involved in such procedures, albeit that the direct negative welfare effects on the donor animal on which the skin biopsy is being performed are mild and can be alleviated.

## Conclusion

Arguments about unfair sporting advantage are unconvincing grounds for considering equine cloning unethical. There is no evidence that eating either cloned horsemeat or cloned meat from other animals poses a public health risk. However, all cloned meat ought to be clearly labelled to enable consumers to select against it on moral grounds. For some people, cloning horses and other species is unethical because cloning goes beyond the limits of how far man ought to interfere with nature and is therefore simply morally repugnant. Such arguments are hard to refute, because they are a matter of moral conscience. However, the concept of ‘an affront to dignity’, which forms part of some moral objections to cloning, seems a weak ethical reason for branding cloning ethical.

Concerns about the health and welfare of recipient animals gestating and giving birth to clones and about the short, medium and long‐term health and welfare of cloned farm animals provide compelling reasons to consider cloning unethical on cost:benefit grounds. What little evidence exists so far suggests that some welfare problems which are prominent in the cloning of farm animals, particularly fetal oversize and dystocia, do not occur with such significance in horses. However, other reported problems, particularly those occurring in equine neonates and foals, render use of the technique ethically dubious. The onus is on all those providing commercial equine cloning services to provide a stronger evidence base for ethical decision making about equine cloning by collating data about the short, medium and long‐term health of cloned horses. This will require collaboration not only between specialist centres, but also with veterinarians who are not specialists in cloning, but who provide healthcare for cloned offspring throughout their lives.

## Author's declaration of interests

No conflicts of interest have been declared.

## Ethical animal research

This paper has been assessed according to the Royal Veterinary College's Code of Good Research Practice (authorisation No 01144).

## Source of funding

The author undertook the research for this article whilst funded by the Wellcome Trust as a Biomedical Ethics Research Fellow.

## Note

1 Animals' needs to express normal behaviours (an interest which should be respected) might be analogous to ‘dignity’
